# A Case Report: Point-of-care Ultrasound in the Diagnosis of Post-Myocardial Infarction Ventricular Septal Rupture

**DOI:** 10.5811/cpcem.2020.4.47073

**Published:** 2020-06-15

**Authors:** Andrew J. Portuguese, Khaled H. Abdulla, Michael Vornovitsky, John DeAngelis

**Affiliations:** * University of Rochester Medical Center, Department of Medicine, Rochester, New York; † University of Rochester Medical Center, Department of Cardiology, Rochester, New York; ‡ University of Rochester Medical Center, Department of Emergency Medicine, Rochester, New York

**Keywords:** Coronary artery disease, mechanical circulatory support, ST-elevation myocardial infarction, ventricular septal rupture

## Abstract

**Introduction:**

Ventricular septal rupture (VSR) is a rare complication of ST-elevation myocardial infarction (STEMI), typically discovered post-revascularization.

**Case report:**

We present the first case of VSR detected on point-of-care ultrasound (POCUS) in the emergency department immediately prior to emergent angiography, with management positively affected by this discovery. The VSR was quickly confirmed via right heart catheterization. Subsequently, hemodynamic stability was achieved using an intra-aortic balloon pump. A delayed surgical VSR repair, with concomitant coronary artery bypass grafting, was implemented for definitive management.

**Conclusion:**

This case highlights the utility of POCUS in a STEMI patient with a suspected mechanical complication.

## INTRODUCTION

Ventricular septal rupture (VSR) is a serious complication of ST-elevation myocardial infarction (STEMI) associated with extremely high mortality. Development of VSR is thought to be mediated by the complete occlusion of a major vessel in the absence of spontaneous reperfusion or collateral circulation. In this regard, VSR is less likely to occur in a patient with a history of symptomatic coronary artery disease (CAD).^1^ Risk factors shown to be independently associated with development of VSR include advanced age, female gender, and chronic kidney disease. Paradoxically, hypertension and diabetes mellitus appear to confer protection, possibly via concentric myocardial hypertrophy and increased collateral circulation, respectively.^2^

Diagnosis of VSR generally occurs within the first week post-MI, typically presenting as a clinical decompensation in patients who received timely revascularization. Whereas the median time from symptom onset to diagnosis is approximately five days, earlier occurrence (≤ two days) has been associated with thrombolytic utilization. In roughly 50% of cases, VSR occurs with total occlusion of the infarct-related artery. Anterior and inferior-posterior infarcts occur with roughly equal frequency. Hemodynamic instability often accompanies VSR. Cardiogenic shock is present in 39% of cases, and cardiac arrest occurs in 6.1% of cases.^2^ Current European Society of Cardiology guidelines recommend immediate echocardiographic assessment when mechanical complications are suspected.^3^ In many cases, particularly in the emergency department (ED) setting, point-of-care ultrasound (POCUS) may be the most rapidly available test to establish this diagnosis.

## CASE REPORT

A 61-year-old man with a history of long-term cigarette smoking (45 pack-years) presented to the ED with acute dyspnea and one week of chest pain. Initial vital signs were as follows: temperature 36°C, heart rate 97 beats per minute, blood pressure 176/100 millimeters of mercury, respiratory rate 30 breaths per minute, and oxygen saturation (SpO_2_) 89%. He required four liters per minute supplemental oxygen to maintain SpO_2_ greater than 94%. An electrocardiogram revealed Q waves and ST-segment elevations in leads II, III, and aVF with reciprocal depressions in leads I, aVL, and V_2_–V_5_ (Image, Panel A), consistent with subacute inferior STEMI. His exam was notable for a harsh holosystolic murmur heard throughout the precordium. The patient’s preliminary laboratory results revealed a lactic acidosis with arterial lactate of 9.2 millimoles per liter (mmol/L) (normal range 0.5–2.2 mmol/L), and a leukocytosis with a white blood cell count of 19.8 × 10^9^/L (normal range 4.2–9.1 × 10^9^/L). His high sensitivity troponin T was 5853 nanograms (ng)/L (normal range 0–21 ng/L).

**CPC-EM Capsule**

What do we already know about this clinical entity?

Ventricular septal rupture (VSR) is a devastating ST-elevation myocardial infarction (STEMI) complication that requires prompt diagnosis and management.

What makes this presentation of disease reportable?

Early discovery of a post-STEMI VSR on point-of-care ultrasound (POCUS) allowed for optimal management and led to a positive outcome.

What is the major learning point?

Immediate echocardiographic assessment should be performed whenever post-STEMI mechanical complications are suspected.

How might this improve emergency medicine practice?

Rapid screening of the unstable STEMI patient using POCUS can help diagnose VSR and facilitate appropriate management.

On POCUS in the ED, parasternal long- (Image, Panel B) and short-axis (Image, Panel C) views revealed a large VSR, and a modified apical four-chamber view (Video) with color flow Doppler confirmed left-to-right flow across the defect (Image, Panel D and Video). Emergent coronary angiography demonstrated a chronically occluded proximal right coronary artery and an 80% proximal- to mid-left circumflex stenotic lesion; no stents were placed. The cardiac index was 1.0 L/minute (min)/squared meter (m^2^) (normal range 2.5–4.2 L/min/m^2^) and the pulmonary-to-systemic flow ratio (Q_p_:Q_s_) was 7.8 (normal ratio of 1), consistent with an extremely large left-to-right intraventricular shunt. Oxygen saturation measurements collected during right heart catheterization (RHC) are shown in the table.

The coronary artery stenotic lesions were not amenable to stenting. Hemodynamic stabilization was achieved with placement of an intra-aortic balloon pump (IABP). On hospital day 10, single vessel coronary artery bypass grafting (CABG) and VSR repair were completed successfully. The IABP was removed the following day. Post-op transthoracic echocardiography (TTE) demonstrated moderately reduced left ventricular ejection fraction (30–39%; calculated at 35%; normal range 55–70%) and trivial left-to-right shunting. His post-op course was uncomplicated, and the patient was discharged home on hospital day 15.

At two-week post-discharge follow-up with his new cardiologist, the patient endorsed improving exertional fatigue and was otherwise asymptomatic. His recovery had been uneventful. He remained abstinent from cigarette smoking.

One month after discharge, repeat TTE and RHC were completed to rule out significant left-to-right shunting. TTE demonstrated an improvement in left ventricular ejection fraction, now only mildly reduced (40–49%; calculated at 44%), and a borderline increase in left-to-right shunting. RHC showed a mild step-up in oxygen saturation from the mixed venous to the right ventricle (53 to 65%, respectively), with a calculated Q_p_:Q_s_ of 1.4, and cardiac index of 2.3 L/min/m^2^ (Table).

## DISCUSSION

Mechanical complications of STEMI are life-threatening and necessitate prompt diagnosis and management. Concerning findings (e.g., acute hypotension, recurrence of chest pain, a new cardiac murmur, pulmonary vascular congestion, or jugular vein distension) should raise suspicion and trigger immediate echocardiographic assessment.^3^ In the ED, POCUS shows promise as a rapid and easily accessible screening tool in the diagnosis of VSR, as well as other mechanical complications (e.g., RV wall rupture and aortic dissection).^4^–^6^ Key sonographic findings of VSR include direct visualization of the defect, blood flow across the interventricular septum, and RV dilation.^7^ Since the defect may not be visible in standard imaging planes, it is necessary to sweep through the interventricular septum and obtain non-standard windows.^5^ Although standard echocardiographic evaluation is highly sensitive (90%) and specific (98%) for the diagnosis of VSR,^8^ the test characteristics of POCUS are unknown, and likely depend on the size and position of the VSR and the ultrasound operator’s proficiency.

Correcting the VSR is essential, with surgical repair representing the gold standard approach. In medically treated patients, the prognosis is grave, with in-hospital mortality ranging from 94–100%.^9^,^11^ Although critical, the optimal timing of VSR repair is not clearly defined. In the immediate period, the fragile necrotic myocardium represents a problematic, technically challenging surgical substrate. Surgical delay is associated with improved 30-day and long-term survival, with an inverse relationship between 30-day mortality and time from diagnosis to repair.^12^ Furthermore, patients who undergo early surgical repair (≤2 days after diagnosis) have markedly worse outcomes compared to those who undergo delayed surgery (>2 days after diagnosis; one-year survival rate 38% vs 64%, p <0.05).^10^ Unfortunately, delay of surgery is often limited by hemodynamic instability.^10^,^13^

The advent and wide availability of mechanical circulatory support (MCS) devices has dramatically altered VSR management. The hemodynamic stabilization afforded by MCS allows for recovery of end-organ injury and serves as a bridge to definitive repair. Current MCS options include IABP, left ventricular assist devices (eg, Impella and TandemHeart), and extracorporeal membrane oxygenation. Currently, European Society of Cardiology and American College of Cardiology Foundation/American Heart Association guidelines suggest using IABP as a stabilizing measure.^3^,^14^ However, there is a paucity of clinical evidence demonstrating the superiority of any one approach. A recent publication by Pahuja et al examined the hemodynamic effects of the above MCS devices in VSR using a computer simulation model. In this model, although no percutaneous MCS completely normalized hemodynamics, pulmonary capillary wedge pressure and left-to-right shunting were worsened by extracorporeal membrane oxygenation and most improved by Impella.^13^

It is uncertain whether revascularization with concomitant CABG at the time of VSR repair improves outcomes. Some studies fail to demonstrate a benefit, instead finding an association between the number of anastomoses and worse mid- to long-term outcomes.^7^ However, it is plausible that the indication for CABG, namely extensive CAD, is the poor prognostic factor rather than CABG itself. By controlling for the severity of CAD using carefully matched cohorts, concomitant CABG at the time of VSR repair has been shown to be associated with improved long-term survival.^15^ This result lends support to the hypothesis that late revascularization is beneficial in this population.

## CONCLUSION

VSR is a rare but devastating complication that typically occurs three to five days post-myocardial infarction and requires definitive surgical repair. When a post-STEMI VSR or other mechanical complication is suspected, immediate echocardiographic assessment should be performed. Our case highlights that this can be accomplished using POCUS. Immediate VSR repair carries a high mortality risk due to the fragility of necrotic tissue. When feasible, a delayed approach enables a more durable repair of scarred tissues, markedly improving outcomes. Performing concomitant CABG at the time of VSR repair is controversial but may improve long-term survival.

## Supplementary Information

Video Modified apical four-chamber view on point-of-care ultrasound. The ventricular septal rupture is directly visualized by sweeping the imaging plane through the interventricular septum. Color flow Doppler demonstrates left-to-right blood flow across the defect.

## Figures and Tables

**Image:**
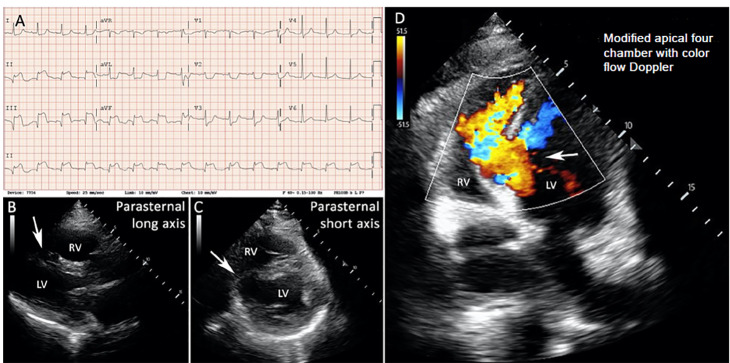
Initial emergency department diagnostics: A) The initial electrocardiogram demonstrates a subacute inferior ST-elevation myocardial infarction; B) Parasternal long; and C) short-axis views reveal an interventricular septal defect; D) A modified apical four-chamber view with color flow Doppler demonstrates a left-to-right shunt across the defect. Arrows point to the interventricular septal defect. *LV*, left ventricle; *RV*, right ventricle.

**Table:** Initial and follow-up right heart catheterization results. Oxygen saturations were measured in the superior vena cava (SVC), high right atrium (hRA), middle right atrium (mRA), low right atrium (lRA), inferior vena cava (IVC), right ventricle (RV), main pulmonary artery (MPA), and arterial blood (SaO2). The calculated Qp:Qs and cardiac index (CI) are listed.

RHC\Chamber	SVC	hRA	mRA	lRA	IVC	RV	MPA	SaO_2_	Qp:Qs	CI (L/min/m^2^)
Initial	31%	39%		39%	13%	86%	88%	97%	7.8	1.0
Follow-up	50%		66%		62%	65%	65%	97%	1.4	2.3

*RHC*, right heart catheterization; *Qp:Qs*, pulmonary-to-systemic flow ratio; *L*, liters; *min*, minute; *m^2^*, squared meter.
